# Impact of Environmental Information Disclosure Policy and Trade on Chinese Paper Industry Environmental Effects

**DOI:** 10.3390/ijerph191811614

**Published:** 2022-09-15

**Authors:** Shuo Feng, Ke Chen

**Affiliations:** College of Economics and Management, Shenyang Agricultural University, Shenyang 110866, China

**Keywords:** environmental information disclosure, trade, environmental effect

## Abstract

While participation in the international division of labor has led to rapid economic development, it has also resulted in pressing environmental issues in China. In the context of “building a resource-saving and environment-friendly society” and the current sustainability requirements, research on the environmental impact of Chinese paper companies from the perspective of Environmental Information Disclosure (EID) policy and trade has not yet reached a consensus. This study constructs an analytical framework for the EID policy impact mechanism and trade on the environmental effects of the paper industry and enterprises. It explores the direct and indirect effects of EID policy and import-and-export trade on the paper industry environmental effects using the Propensity Score Matching and Difference-in-Differences (PSM-DID) model. EID positively impacts the pollution reduction of enterprises mainly through the technical effect. Export trade positively impacts the reduction of enterprises’ emissions through the technology effect. However, the demand of the international market increases the pollution from the paper industries, which has a negative impact. Importing will enable enterprises to obtain greater price advantages which can alleviate and transfer the costs brought by EID. This study analyzes the impact of trade on the environmental effects of Chinese paper enterprises and identifies the impact of China’s EID policy and trade on enterprises’ pollution emissions. It provides a theoretical and practical foundation for the Chinese government to formulate environmental and trade policies.

## 1. Introduction

In addition to rapid economic growth, trade liberalization has been accompanied by a steady deterioration of the natural environment resulting in international trade disputes related to environmental concerns. The continued growth of international trade has led to increasing demand for environmental resources. In contrast, the supply of environmental resources is limited; thus, there is a conflict between free trade and environmental protection. The environmental effects of trade are controversial in academia, and the existing literature has developed three distinct views: harmful, beneficial, and complex theories. Daly and Goodland (1994) are proponents of the harmful theory and were the first to argue that free trade can harm the environment [1]. Their core arguments are that trade growth has expanded the world economy and greatly increased the input of factors, resulting in increased pollution emissions, thus causing environmental harm. The benefit theory is represented by Anderson et al. (1992), whose argument is that trade does not cause environmental problems [2]. They argue that free trade is not impacting the environment but can contribute to environmental protection. Their main idea is that free trade helps to achieve the optimal allocation of environmental resources on a global scale and facilitates the elimination of trade-distorting policy measures, which can be detrimental to environmental protection. Grossman and Krueger (1993), studying the environmental effects of the North American Free Trade Agreement (NAFTA), decompose the environmental effects of trade into three parts: scale, technology, and composition effect, respectively [3]. The complexity of trade environmental impact is the sum of those effects. Antweilei, Copeland, and Taylor (2001) established the General Equilibrium Model (ACT), which centralizes many influencing factors within an analytical framework [4]. The establishment of the ACT model has given a mathematical basis for the decomposition of trade-induced environmental effects.

The public’s awareness of environmental protection has increased with the improvement of living standards [5]. To win the “promotion tournament,” local governments focus on implementing environmental regulation policies. Environmental regulation aims to improve the environmental quality of enterprises by reducing their waste emissions through policy constraints [6]. According to the Porter hypothesis, environmental regulation brings an “innovation compensation effect” that offsets the environmental regulatory costs for firms [7]. Environmental regulation can be measured by the construction of pollutant emission intensity indicators [8], the proportion of pollution investment [9,10], and the number of environmental administrative penalty cases or environmental regulations [11,12]. Most of the measurement instruments are rather one-sided. Since 2009, some cities have been involved in the Environmental Information Disclosure initiative (EID). EID reflects the intensity of environmental regulation by local governments through the Public Disclosure of Information on Pollutant Information Transparency Index (PITI). The PITI comprises five primary and eight secondary indicators and is the most comprehensive measure of environmental regulation variables. Hence, EID is essentially an environmental regulation policy.

Environmental regulation indirectly affects the companies’ environmental impact through its regulatory effect on trade. However, the impact of environmental regulation on trade is still debated. One view supports that environmental regulation hurts trade. This view considers that the imposition of more stringent environmental regulations in a country increases firms’ production costs, reducing the international competitiveness of their products [13]. The contrasting view, represented by Porter et al. [7], is that harsher environmental regulations increase the international competitiveness of a country’s pollution-intensive industries. Appropriate environmental regulations stimulate innovation and improve environmental quality. A third view suggests that the effect of environmental regulation on reducing or increasing international competitiveness depends on the combined results of two variables environmental costs and technological innovation. It further took into account the influence of additional factors and was represented by Alpay and Savas (2001) [14].

With China’s inclusion in the World Trade Organization (WTO), research on the effects of trade and the environment has gradually increased. Still, it is mainly focused on the heavy industry sector. Paper production enterprises have their unique operational characteristics. Due to data availability, there has been less focus on Chinese paper production companies. The limited use of microdata mining has resulted in insufficient analysis of the environmental effects of trade on paper production enterprises. The environmental impact mechanisms are still unclear, and no exploration of the environmental effects of paper enterprises from an EID policy and trade perspective has been undertaken. This study used panel data from the China Industrial Enterprise Database, the China Industrial Enterprise Pollution Database, and the China Customs Enterprise Database. Additionally, selected paper-producing enterprises with high water pollution were considered. The PSM-DID research method explored the direct effects of EID policy and trade on enterprise pollution emissions. The interactions between EID policy and trade variables were used to explore the indirect effects of environmental regulations on the industries’ pollution emissions through trade.

In the context of comprehensively promoting market-oriented reforms, the government and academia need to address the formulation of suitable environmental regulation policies and trade policies for paper enterprises in China as a result of the new trade environment. The innovation of this study has three aspects. First, the impact of the EID policy on pollution reduction of paper enterprises is explored. Secondly, the ACT theory is used to analyze the three paths of trade to enterprise pollution reduction. Third, it explores the moderating effect of environmental regulation on trade. The marginal contribution of this study is that the PSM-DID research method is used to introduce environmental regulation into the discussion of trade environmental effects. This study enriches the paper industry trade research by discussing the trade impacts on the environment and analyzing the intensity and direction of effects. Combined with the study’s findings, it will provide important theoretical background and a realistic basis for developing an active environmental and trade policy for the paper industry in China.

## 2. Theoretical Background and Research Hypotheses

### 2.1. The Impact of Environmental Regulation on the Environmental Effects of Paper Enterprises

The environmental regulation on pollution reduction directly impacts companies through its effect on the labor and capital factors. There are varied outcomes on the impact of environmental regulation on the labor factor (mainly refers to the labor force), as environmental regulation can have a crowding-out effect on the labor factor, which inhibits employment [15]. On the contrary environmental regulation can result in an absorption effect on labor (mainly refers to the employment created), which promotes employment [16]. In terms of theoretical analysis, Morgenstern et al. (2002) carried out a breakthrough analysis by decomposing the impact of environmental regulation on labor factors into the “demand effect”, “cost effect”, and “factor substitution effect” [17].

According to the analyses by Morgenstern et al. (2002) [17], the “demand effect” of environmental regulation on labor factors will lead to higher prices of products, reduce the competitiveness of products, have a crowding out effect on labor factors, and then affect the output of enterprises. This will affect the scale of enterprises’ pollution emissions. Thus the “demand effect” of environmental regulations on labor factors impacts the enterprises’ pollution emissions through scale effects. The “cost-effective” of environmental regulation on labor factors refers to the fact that when the intensity of environmental regulation and the return of labor factors are low, the environmental regulation enterprises will pass on the cost to the production cost. Thus, they will reduce the input of labor factors by compressing the scale of production. When the environmental regulation intensity and the return of labor factors are higher, environmental regulation will force enterprises to carry out environmental protection technology research and development activities. This will then produce the growth of labor demand that matches such activities. Therefore the “cost effect” of environmental regulations on labor factors affects the enterprises’ pollution emissions through scale or technical effects. The “factor substitution effect” results in enterprises being pressured by environmental regulation to increase labor factor inputs related to environmental technology. This might also result in the rection of labor factor inputs involved in the pollution sector. Therefore, the “factor substitution effect” affects the pollution emissions of enterprises through the technology effect or scale effect.

The impact of environmental regulation on capital factors is also inconsistent. On the one hand, environmental regulation can hurt capital factors, based on the pollution heaven theory. This theory suggests that firms will move to areas where environmental regulations are weaker and may respond to environmental regulations by reducing the size of the polluting sector. This way, the impact of environmental regulations on capital factors affects firms’ pollution emissions through scale and structural effects. On the other hand, environmental regulation can positively impact capital factors, based on the Porter Hypothesis through the effect of technology [7].

Considering all the above, the impact of environmental regulation on pollution emissions is achieved through changes in factor inputs in three ways: scale, technology, and structure effects. Based on the characteristics of the paper-producing enterprises, hypothesis 1 is proposed as follows.

**Hypothesis** **1** **(H1).** *EID policy has negative effects on the pollution emissions of Chinese paper enterprises*.

In essence, the EID policy is an environmental regulation, which includes five first-level indicators of “supervision information”, “self-monitoring”, “interactive response”, “emission data” and “environmental impact assessment (EIA) information”. It is the most comprehensive environmental regulation policy at present. Under the pressure of environmental regulation, paper enterprises will inevitably take pollution reduction measures.

### 2.2. The Impact of Trade on the Environmental Effects of Paper Enterprises

Antweilei, Copeland, and Taylor (2001) developed a general equilibrium model, called the ACT model, that brings many influencing factors within a single analytical framework [4]. It decomposes the root causes of the environmental pollution problems caused by trade into three categories: scale, structural, and technological effects. In specific empirical studies, the heterogeneity of industries and stages of industry development must be considered. Therefore, the findings of these three effects are inconsistent in different research settings.

Export trade mainly affects scale, structure, and technology through the “demand effect” in international markets and the “learning-in-export effect.” Import trade affects scale, structure, and technology mainly through the cost effect mechanism.

Unlike industrial chemical companies, which have been the focus of most of the literature, paper-producing companies usually operate in international markets to absorb production capacity, improve technology and efficiency, and use foreign intermediate raw materials to produce end products. When paper enterprises export their products, their output is expanded, and the scale effect has a positive effect. Still, exporting trade can lead to higher environmental standards and technology in developed countries, resulting in reduced pollution emissions, i.e., a technology effect. At the same time, paper enterprises may adapt to the international market by enriching their product range. This leads to changes in their product range indirectly impacting pollution emissions, i.e., a structural effect. Paper-producing enterprises mainly import raw materials. The domestic price of raw materials is usually higher than the international price, so importing raw materials with lower prices will stimulate production. Thereby the pollution emissions will be increased, i.e., a scale effect. So, hypothesis 2 is proposed as follows.

**Hypothesis** **2** **(H2).** *Export trade has negative effects on the pollution emissions of paper enterprises, and import trade has positive effects on the pollution emissions of paper enterprises*.

### 2.3. The Impact of Environmental Regulation and Trade Interactions on the Environmental Effects of Paper Enterprises

The analysis of the environmental regulation impact on trade is mainly based on the factor endowments theory and the Porter hypothesis theory [7,18]. The analytical conclusions of the two theories, however, are the opposite. Research based on factor endowment theory argues that environmental regulation harms trade. The core of factor endowment theory is that increasing the intensity of environmental regulation will increase the production costs of enterprises. This will weaken the international competitiveness of domestic products and eventually cause domestic trade to be transferred to other countries, resulting in the “trade transfer effect of environmental regulation [19]. Research based on the Porter hypothesis theory suggests that environmental regulation positively impacts trade. Increasing environmental regulation intensity can promote production technology innovations and improve enterprises’ production efficiency, thereby increasing comparative product advantage. Existing studies have shown that the environmental regulation innovation effect can compensate for the cost effect, forming the “trade innovation effect of environmental regulation” [20,21]. So, hypothesis 3 is proposed as follows.

**Hypothesis** **3** **(H3).** *The EID policy negatively impacts paper enterprises’ pollution emissions through trade*.

Hypothesis 3 is proposed based on hypothesis 2. Based on the characteristics of paper-producing enterprises, trade can enable them to obtain a larger market and lower raw material prices. However, under the pressure of environmental regulations, the technical effect may be greater than the scale and structural effect, respectively.

## 3. Materials and Methods

### 3.1. Data Sources

The data used in this study were panel data constructed by China Industrial Enterprise Database, China Industrial Enterprise Pollution Database, and China Customs Enterprise Database. The data were obtained from EPS micro-enterprise database, with data published by China Industrial Enterprise Database from 1998 to 2015, data published by China Industrial Enterprise Pollution Database from 1998 to 2014, and data published by China Customs Enterprise Database from 2000 to 2016. Most of the financial information at the enterprise level was obtained from the China Industrial Enterprise Database, the detailed information on enterprise trade was obtained from the China Customs Enterprise Database, and the information on enterprise pollution emissions was taken from China Industrial Enterprise Pollution Database. Therefore, this study’s panel data period was from 2000 to 2014.

### 3.2. Data Processing Process

**Figure 1:**
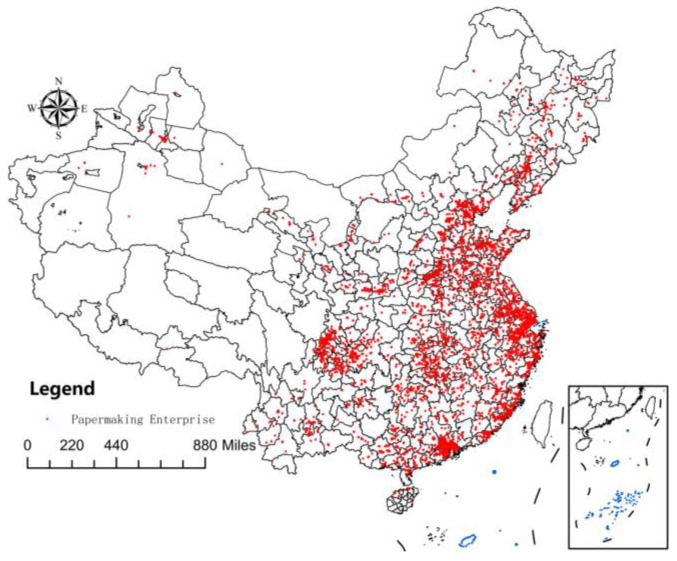
Spatial distribution of samples.

To construct the required panel data, it was necessary to match the three micro-databases effectively. Although the EPS database can provide matched data, there is a serious problem with sample selection in their data form. Therefore, in the first step, the methodology by Brandt et al. (2012) was used to merge the databases of Chinese industrial enterprises into panel data [22]. At the same time, the data were cleaned up according to the method of Nie et al. (2012) [23]. The second step was constructing basic enterprise panel data from China Industrial Enterprise Database and China Industrial Enterprise Pollution Database. According to Tian and Yu (2013), the third step matches the Chinese customs enterprises’ database [24]. As numerous variables were missing in the 2010 data in China’s industrial enterprise database, the year 2010 was removed from the study. Finally, the enterprises with an industry classification code of 22 are selected, representing the paper industry. In this way, a total of 24,092 enterprises were obtained. Among them, state-owned enterprises accounted for 13.71%, private enterprises accounted for 55.92%, Chinese foreign joint ventures accounted for 7.31%, wholly foreign-owned enterprises accounted for 5.29%, and other types of enterprises accounted for 17.77%. Enterprises producing machine-made paper and paperboard accounted for 70.87%, enterprises producing paper and paperboard containers accounted for 13.34%, and enterprises of other types accounted for 15.79%. The spatial distribution of samples is shown in Figure 1 [25].

### 3.3. Variable Selection

(1) the explained variables. According to the research content and theme, the enterprises’ environmental pollution is measured by the total industrial water consumption logarithm. During sample investigation, some indicators of pollutant emission, such as sulfur dioxide, but as many such samples are missing, this leads to inaccurate estimations. At the same time, an important characteristic of paper-producing enterprises is that they depend on water resources and need to consume a lot of water during production. Although industrial water can’t fully reflect enterprises’ pollution situation, it reflects enterprises’ production efficiency to some extent. In view of this index imperfection, when calculating the robustness regression, the logarithmic index of total industrial water consumption is replaced by the logarithmic index of total industrial wastewater outflow. The total industrial wastewater discharge is one of the main statistical indexes of the China Environmental Statistics Yearbook, and can estimate enterprises’ pollution scale and environmental effects. The data of the above two variables were obtained from the pollution database of China’s industrial enterprises. We used the logarithm of the data to reduce the error caused by heteroscedasticity and to make the regression coefficient have economic significance and the coefficients between variables comparable.

(2) Explanatory variables. The explanatory variables were mainly divided into three parts. EID policy variables, trade variables, and control variables.

Given the policy variables of EID, most previous studies divided environmental regulation into three categories: administrative, market, and public participation [26]. Most papers only studied one aspect, but few comprehensive studies were made on all three aspects. Moreover, the construction of indicators was straightforward, and it wasn’t easy to measure the real level of environmental regulation. The EID policy in this study was measured by PITI, which scores the level of information disclosure of pollution sources in cities through a certain evaluation mechanism [27]. In 2008, the Institute of Public and Environmental Affairs (IPE), a domestic environmental protection NGO, and the natural resources defense council (NRDC) jointly released some Chinese cities’ PITI. The index evaluation includes five first-level indicators: supervision information, self-monitoring, interactive response, emission data, and environmental impact assessment (EIA) information. Eight second-level indicators are also integrated, namely, daily supervision information and centralized remediation information of pollution sources, audit information on cleaner production, comprehensive evaluation information of enterprises’ environmental impact, acceptance of public letters and complaints, acceptance of EIA documents and their results, information on sewage discharges, and applications disclosure. Each index is quantitatively evaluated through four dimensions: systematicness, timeliness, completeness, and friendliness. Therefore, they can comprehensively reflect the intensity of local government’s environmental regulation on enterprises, and the data comes from the Research Report of PITI.

Secondly, according to trade variables, it is divided into two categories: exports and imports. As the export and import data provided by China Customs Enterprise Database are counted in the form of orders, they need to be aggregated on an enterprise basis. The trade value after aggregation is denominated in US dollars and needs to be converted into local currency according to the average exchange rate at the year of trade. It was converted to the price level of the base period in 2000 through the Industrial price index. Finally, the logarithm is taken, so the regression coefficient has economic significance.

Finally, the control variables are divided into two categories. The enterprise-level control variables comprise one category. Using the methodology of Cheng et al. (2021) for reference, the enterprise scale lnSize was calculated by the logarithm of the number of employees, and the asset specificity lnSpe was calculated by the logarithm of the ratio of fixed assets to total assets [28]. Using the methodology of Ma et al. (2021), the asset-liability ratio lnDe was calculated by dividing the total liabilities of an enterprise by the total assets and then taking the logarithm [29]. The capital intensity of enterprises lnKL was estimated by the logarithmic value of the ratio of the actual total fixed assets of enterprises to the number of employees, according to Wei et al. (2021) [30]. Accurate estimation of enterprise total factor productivity is an important basis for studying enterprise behavior. Although OP and LP estimation methods can, to a significant extent, solve the problem of synchronicity error, Ackerberg et al. (2015) suggested that such methods have the problem of function dependence, which can’t be effectively estimated [31]. The problem of functional dependence can be solved by adding free variables into the intermediate input function and adopting nonparametric estimation in the first step of estimation [31]. On the basis of the OP method, the method of adding free variables into the intermediate input function is defined as the OP-ACF method. Therefore, our study adopted the OP-ACF method to estimate enterprise total factor productivity. All the variables related to the price are converted to a price level based on the year 2000 price index to eliminate the influence of the price factor. The second category of control variables is variables at the regional level. There is heterogeneity in the development level, industrial structure, and human resources among cities. To control the impact of these factors, according to the literature, we summarized the marketization index, industrial structure (the ratio of the secondary industry to the tertiary industry), the proportion of loans to gross domestic product (GDP), the number of college students, per capita GDP and the proportion of Foreign Direct Investment (FDI) to GDP. However, serious collinearity problems will occur when the proportion of loans to GDP and the proportion of FDI to GDP are added to the regression model. Therefore, we eventually adopted four control variables, namely marketization index (MI), industrial structure (II), human resources (St), and per capita GDP, to control four influencing factors, namely marketization degree, industrial structure, human resources, and economic development level, respectively. Making variables took the logarithm. Among them, the market index data was calculated by Fan et al. (2011) method, and other variables were obtained from the Yearbook of Regional Statistics [32]. The statistical analysis of variables is shown in Table 1. From the total industrial water consumption W, after the implementation of the EID policy, the average value of the treatment group is 4,383,100, and the average value of the control group is 4,941,318. It can be found that the average value of the treatment group is significantly smaller than that of the control group, which indicates that the EID policy has played a limiting role in the total industrial water consumption of enterprises. From the industrial wastewater discharge W1, after the implementation of the EID policy, the average value of the treatment group is 1,274,099, and the average value of the control group is 1,025,522. The two groups of data not only have significant differences intuitively, but also pass the t-test, and the effect of EID policy on pollution reduction of paper enterprises is statistically guaranteed.

### 3.4. Model Setting

Here we used the multi-period DID model to identify the direct impact of EID policy on enterprise pollution and its indirect impact on enterprise emission reduction through trade. The EID policy variable was represented by PITI, which can be set as a continuous or discrete variable. The processing variable is set as whether an enterprise is in the published PITI area. To eliminate the biased estimation results caused by sample self-selection, propensity score matching (PSM) was used to process the samples [33]. Taking the total industrial water consumption of each enterprise as the response variable, the benchmark multi-period DID Model (1) was obtained as follows:

(1) $$\ln W_{it}=\alpha_{0}+\alpha_{1}DID_{it}+\alpha_{2}\ln ex_{it}+\alpha_{3}DID_{it}*\ln ex_{it}+\alpha_{4}\ln im_{it}+\alpha_{5}DID_{it}*\ln im_{it}+\sum_{i=1}^{n}\beta_{i}X_{it}+\mu_{it}$$

*lnW_it_* indicates the total industrial water consumption of enterprise *i* in year *t*; The core explanatory variable *DID_it_* of the multi-period DID model is a virtual variable that varies from enterprise to enterprise. When enterprise *i* is affected by EID policy in t year, it receives a value of 1; otherwise, it receives a value of 0. To avoid problems caused by missing related variables, a series of control variables *X_it_* were added to the model, including enterprise size (*lnSize*), asset specificity *(lnSpe*), asset-liability ratio (*lnDe*), enterprise capital intensity (*lnKL*), enterprise total factor productivity (*opacf*), enterprise age (*year*), marketization index (*MI*), industrial structure (*II*), human resources (*St*) and per capita GDP.

Besides the above control variables, since the model is a panel data model, time fixed effect and space fixed effect are added to the model. These control the estimation error caused by missing variables that change with time and space. To reduce serial correlation, the standard error of clustering to the enterprise level is adopted in the regression process.

## 4. Results

### 4.1. Impact of EID Policy and Trade on the Environmental Effects of Paper-Producing Enterprises

Based on Formula (1), this paper regresses enterprises’ total industrial water consumption *lnW_it_* against *DID_it_*, export trade, and various control variables (Table 2). Considering that in regression analysis, the coefficient and standard error of the core explanatory variables are often affected by the selected control variables and fixed effects, improper selection of any aspect may cause bias in the estimated results and lead to unreliable results. Therefore, to strengthen the reliability of the regression analysis results, we investigated the influence of two-way fixed effects, the standard error from clustering to enterprise level, and the introduction of different control variables on the research results. Table 2(1)–(9) add control variables step by step under the two-way fixed effects of time and space. As shown by the results, the gradual addition of control variables resulted in a lowering of the coefficient of the EID policy variable *DID_it_*, with the significance level also decreasing. However, the impact of the EID policy on the industrial water consumption of enterprises was significantly negative (*p* ≤ 0.05). The regression coefficient was −0.197, which indicates that the implementation of the EID policy can effectively alleviate the total industrial water consumption of paper-producing enterprises and further provides evidence to support the direct effect of the EID policy on pollution reduction (Hypothesis 1).

The estimated result of export trade *lnex* was significant at *p* ≤ 0.01, and the coefficient reached was −0.08. Thus, the paper-producing enterprises’ pollution will be reduced by 0.078% for every 1% increase in exports. The regression coefficient of import trade *lnim* reached 0.033 when the control variables were gradually added (*p* ≤ 0.05). This indicates that imports will increase the pollution emitted by paper-producing enterprises. For every 1% increase in imports, the industrial water consumption of paper-producing enterprises will increase by 0.033%. It provides evidence to support export trade has negative effects on the pollution emissions of paper enterprises, and import trade has positive effects on the pollution emissions of paper enterprises (Hypothesis 2). Export trade decreases pollution emissions, while import trade increases pollution emissions.

By introducing the two interactive items of EID policy variables and trade, *DID_lnex* and *DID_lnim* are used to measure the moderating effect of EID policy on trade. When the coefficient of interaction term and the coefficient of trade variable are in the same direction, it indicates that EID policy has a positive moderating effect on trade. On the contrary, it shows that EID policy has a negative moderating effect on trade. The *DID_lnex* coefficient of the interaction between EID policy and export trade was −0.108 (*p* ≤ 0.01) and is in the same direction as the export trade influence coefficient. It shows that EID policy has a positive moderating effect on export trade’s environmental effect. If the paper-producing enterprises increase their exports by 1% on average, the industrial water consumption of enterprises will decrease by 0.078%. Under the EID policy’s adoption, enterprises’ industrial water consumption will decrease by 0.186% for every 1% increase in exports. Thus, it provides evidence to support part of hypothesis 3. The moderating effect of EID policy on import trade *DID_lnim* has a coefficient of 0.108 (*p* ≤ 0.01) and is in the same direction as the import trade coefficient. It indicates that, like export trade, EID policy also has a positive moderating effect on import trade. However, unlike export trade, both import trade and EID policy’s moderating effect on import trade aggravate the environmental pollution of enterprises. It does not provide evidence to support all of Hypothesis 3.

### 4.2. Robustness Tests

According to previous literature, the characteristics of this study, and the data availability, five robustness tests were conducted: (1) Parallel trend test. The prerequisite of DID application is that the samples of the treatment group and the control group have the same development trend before the policy implementation, so a parallel trend test is an essential part of using the DID method; (2) Placebo test. Because this study’s sample data covers a long period, other policy factors may influence the treatment group and the control group. To exclude the influence of other policies on the research results, a placebo test is needed; (3) A multi-period and multi-individual DID to test the robustness and accuracy of EID policy regression results; (4) Replace the explained variables and replace the total industrial water consumption with the industrial wastewater discharge of paper-producing enterprises. Wastewater discharge is a more direct and accurate measurement of enterprise pollution and thus examines the robustness of variables; (5) Taking the lag period of the explained variable as a new explained variable can alleviate the endogenous problem caused by the causality between independent variables and dependent variables. Concurrently, it is beneficial to investigate the dynamic effect of EID policy and import and export trade variables on enterprises’ pollution. The main purpose of test (1) is to check whether the model meets the preconditions for using the Difference-in-Differences method. The main purpose of test (2) is to analyze whether the model is disturbed by other treatment effects. Tests (3)–(5) are all to verify the robustness of the model. However, the focus of the three tests is different. The essence of test (3) is to examine the reliability of the regression results in the case of changing the treatment variable, and the essence of test (4) is to examine the robust-ness of the regression results in the case of changing the explained variables. Test (5) consider the estimation bias caused by the endogenous problem.

#### 4.2.1. Parallel Trend Test

**Figure 2:**
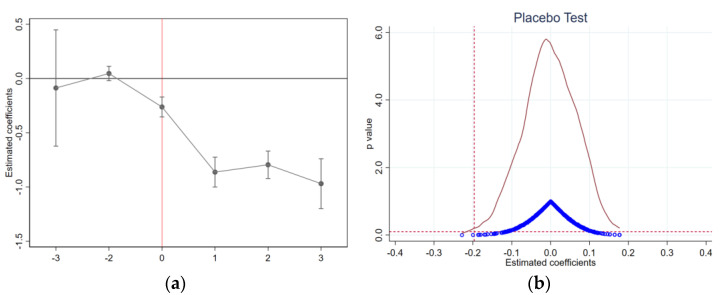
Parallel trend test (**a**) and placebo test (**b**). **Note:** In the parallel trend test (**a**), the horizontal axis indicates the period before and after EID policy implementation, 0 represents the current period, 1 represents one year after implementation, and −2 represents two years before policy implementation. In multi-period DIDs, to avoid collinearity, period −1 should be excluded, in which the solid point represents the value of the coefficient, and the extension line represents the confidence interval of 95%. In the placebo test (**b**), the blue circles represent the coefficient calculation results of 500 groups of virtual policy variables, and the red curve represents the distribution of coefficients. The red horizontal dotted line illustrates the significance level of 10%, and the value of coefficients in benchmark regression is illustrated by the red vertical line.

The premise of the DID method is that the data satisfy the parallel trend hypothesis. According to the design of this study, the sample enterprises that implement the EID policy should be in parallel with those not affected by the policy in terms of total industrial water consumption. Thus, the treatment group should have the same trend as the control group before implementing the treatment effect. To eliminate the influence of sample self-selection, Figure 2a illustrates the dynamic change of the estimated coefficient of the samples after PSM treatment. Before the policy implementation (on the left side of the red vertical line), the coefficient was not significant. In the current implementation plan period, the EID policy significantly negatively impacts enterprises’ total industrial water consumption, suggesting that the control group and the treatment group meet the parallel trend test.

#### 4.2.2. Placebo Test

The above-mentioned DID model controls the fixed effects and the main factors that may lead to the non-random pollution effect of enterprises and has passed the parallel trend test. However, theoretically, it still cannot eliminate the interference of missing variables and other issues in the regression results. The core idea of the DID placebo test is to calculate estimates using a fictitious treatment group. Suppose the coefficient of the “pseudo-policy dummy variable” is significant under fictitious circumstances. In that case, the estimation of the benchmark model result is likely to be biased, and the explained variable is likely to be influenced by other policies or random factors. We further carried out the placebo test in this study to solve the above problems. Randomly selected enterprises were set as the treatment group, which was repeated 500 times to generate 500 groups of random samples. Regression analysis was carried out on each group of random samples, and the estimated results of 500 policy changes on pollution emissions of enterprises were obtained. Figure 2 (right) illustrates the probability density distribution of this set of regression coefficients (red curve). Only the coefficient estimated by individual random simulation is significant at (*p* ≤ 0.1) (horizontal red dotted line). At the same time, the probability density distribution chart reaches its peak near 0. This indicates that most of the coefficients are near 0, and thus have no effects, which is in line with the expected effect of the placebo test.

#### 4.2.3. Multi-Period and Multi-Individual DID

Multi-period DID requires that the processing variables are 0–1 discrete variables. However, according to the actual data, the pollution source information disclosure index PITI is a continuous variable, which enables to conduct of multi-period and multi-individual DID research. PITI changes every year, reflecting the change in local government’s environmental regulation intensity. Multi-period and multi-individual DID directly use the PITI original data, which has more economic meaning and can provide convenience for the follow-up study of EID policy intensity. The regression results are shown in Table 3(1). We replaced the processing variables in Model (1) with the PITI raw data and defined it as an *Index*. To avoid the collinearity caused by the interaction of EID policy and trade, the EID policy remained in a discrete form. From the variable *Index* regression results, the coefficient was calculated at −0.008. Although the influence degree was very low, the significance level was very high (*p* < 0.01), and the robustness of all variables is guaranteed.

#### 4.2.4. Replace the Explained Variable

In China’s industrial enterprise pollution database, the total amount of industrial wastewater discharged by paper-producing enterprises is recorded in addition to the values of the total amount of industrial water used. It is more appropriate to use the total amount of industrial wastewater as the explained variable to measure enterprises’ pollution emissions from enterprise management’s perspective. However, the sample integrity of the total amount of industrial water *lnW* is better, so the total amount of industrial water is used in the benchmark regression model. In this part of the robustness test, the explained variables are replaced by *lnW1* the industrial wastewater discharge. The PSM-DID estimation method is used as before, and the time and space two-way fixed effect and clustering standard error are used for the regression analysis. The results are shown in Table 3(2). After replacing the explained variables, the results of PSM-DID are consistent with those in benchmark regression, which proves that the conclusion of benchmark regression is very robust.

#### 4.2.5. The Variable to Be Explained Lags behind

Although DID regression technology can eliminate the endogenous problem of environmental regulation variables and PSM technology can eliminate problems caused by sample self-selection, it still can’t ameliorate the causality between trade variables and other control variables and the explained variables. To avoid the estimation bias caused by the possible endogeneity, we used the lag 1 variable *L.lnW* as the new explained variable. As the lag period can’t affect the independent variables of the current period, the dependent variables of the lag period can alleviate the endogenous problems caused by mutual causality and make the estimation results more accurate. Table 3(3) lists the results of the analyses. The results were very stable when the explained variables were delayed by one period.

### 4.3. Mechanism Path Test

Theoretically, the impact of EID policy and trade on environmental effects can be divided into three aspects, namely, scale effect, technical effect, and structural effect. To explore the influence of EID policy and trade on these effects, the explained variables were replaced by three variables, using the method of Chen (2021) as a reference [34]. The logarithmic *lnPd* of gross industrial output value represented the scale effect, the logarithmic *lnPol* of the ratio of total industrial water consumption to gross industrial output value represented the technical effect, and the logarithmic *lnPer* of the ratio of [(main business income to total income)+1] represented the structural effect. Technical effect *lnPol* is constructed by the ratio of pollution emission to output. Unit pollution emission can reflect production efficiency and is a measure of technology. The structural effect *lnPer* is constructed by the ratio of the main business income to the total income. By mining the change in the ratio of the main business income to the total income, we can examine the business scope and product structure of the enterprise. Therefore, we use *lnPer* to examine the impact of the structural effect.

The specific regression model is as follows.

(2) $$\ln Pd_{it}=\alpha_{0}+\alpha_{1}DID_{it}+\alpha_{2}\ln ex_{it}+\alpha_{3}DID_{it}*\ln ex_{it}+\alpha_{4}\ln im_{it}+\alpha_{5}DID_{it}*\ln im_{it}+\sum_{i=1}^{n}\beta_{i}X_{it}+\mu_{it}$$

(3) $$\ln Pol_{it}=\alpha_{0}+\alpha_{1}DID_{it}+\alpha_{2}\ln ex_{it}+\alpha_{3}DID_{it}*\ln ex_{it}+\alpha_{4}\ln im_{it}+\alpha_{5}DID_{it}*\ln im_{it}+\sum_{i=1}^{n}\beta_{i}X_{it}+\mu_{it}$$

(4) $$\ln Per_{it}=\alpha_{0}+\alpha_{1}DID_{it}+\alpha_{2}\ln ex_{it}+\alpha_{3}DID_{it}*\ln ex_{it}+\alpha_{4}\ln im_{it}+\alpha_{5}DID_{it}*\ln im_{it}+\sum_{i=1}^{n}\beta_{i}X_{it}+\mu_{it}$$

The above three regression models examine the three action paths of EID policy and trade on enterprise pollution emissions: scale effect, technical effect, and structural effect. See Table 4 for regression results.

#### 4.3.1. Scale Effect

Table 4(1) lists the verification results of the scale effect. EID policy positively impacts the enterprise scale, but the impact intensity was very low. The regression coefficient was only 0.004, and the significance level was also very low (0.05 < *p* < 0.1). From the perspective of export trade *lnex*, the regression coefficient is −0.001, which is significant at 10%. This shows that export trade has a negative impact on the scale of enterprises. For every 1% increase in exports, the gross output value of paper-producing enterprises decreased by 0.001% on average. EID policy had a negative moderating effect on export trade, and its coefficient was 0.001, (*p* ≤ 0.1). This indicates that under the stimulation of EID policy, for every 1% increase in export trade, the GDP of paper-producing enterprises will increase by 0.001% on average. Import trade *lnim* can promote the GDP of enterprises. For every 1% increase in imports, GDP will increase by 0.001%. The significant level of scale effect of import trade was very high, with *p* ≤ 0.01. EID policy has a negative effect on import trade, with a −0.001 coefficient. Although increasing imports can result in a greater cost advantage, the final product will still produce pollution. Thus, under the constraint of environmental regulations, paper-producing enterprises will reduce the gross production value and scale of production.

#### 4.3.2. Technical Effects

Table 4(2) lists the verification results of technical effects. The influence coefficient of EID on *lnPol* was −0.119, *p* ≤ 0.01. The composition of *lnPol* shows that EID policy can promote enterprises’ green production technology level. The regression coefficient of export trade *lnex* was −0.041 (*p* ≤ 0.01). For every 1% increase in export trade *lnex*, the technical level increases by 0.041%. Thus, the EID policy has a positive regulatory effect on export trade. The interaction coefficient between EID policy and export trade is −0.026 (*p* ≤ 0.1). It shows that under the stimulation of EID policy, the technical effect of export trade will be increased. Thus, under the influence of environmental regulation, the total effect of export trade on technological progress is −0.067. From the *lnim* variable of import trade, the regression coefficient of import trade is 0.024 (*p* ≤ 0.01). The moderating effect of the EID policy on import trade is positive, and the coefficient of its interaction is 0.045 (*p* ≤ 0.01)

#### 4.3.3. Structural Effect

Table 4(3) lists the verification results of structural effects. EID policy has no significant impact on the structural effect. The regression coefficient of export trade *lnex* was 0.001 (*p* ≤ 0.05). It indicates that export trade will strengthen the main business of enterprises and weaken their ability to expand product categories. Under the strong demand of the international markets, enterprises will lock in the core products to make full use of the scale effect, thus losing the benefits of economies of scope. The moderating effect of EID policy on export trade failed the T-test. The moderating effect of import trade and EID policy on import trade was also insignificant, indicating that import trade will not interfere with the decision-making of enterprise product structure management.

## 5. Discussion

Usually, enterprises will respond and adapt to the government’s environmental regulations [6,35]. This paper finds that the EID policy has a restrictive effect on the pollution emissions of paper-producing enterprises, thus a positive effect on pollution reduction. This is consistent with the research conclusions of Hu and Li (2020), and Shi et al. (2021) [36,37]. EID policy mainly affects the scale effect, technical effect, and structural effect of the pollution emissions of paper-producing enterprises by influencing the input of labor and capital. From the scale effect perspective, EID policy positively impacts enterprises. From the economic theory perspective, from the micro-enterprise level, EID policy should hurt the production scale of enterprises. A reasonable explanation is that under the implementation of the EID policy, the technological level of enterprises is improved, which leads to the expansion of production scale. The technical effect supports this. The production scale of the enterprise should have been reduced under the constraint of the EID policy, but the production scale of the enterprise has increased slightly under the intervention of the technical effect, which indicates that the impact of the EID policy on the technical effect is greater than the scale effect, and the technological progress makes up for the loss of the scale reduction. Therefore, the comprehensive impact of the EID policy on the scale effect of the enterprise is increased, When the EID policy can effectively improve the green production technology level of enterprises, there is no need to worry that environmental regulation will limit the production scale of enterprises. From the technical effect point of view, EID policy positively impacts the technical effect of paper-producing enterprises, which supports the Porter hypothesis [7]. Hu and Li (2020) suggest that technological progress in emission reduction is important when studying the mechanism of EID’s influence on industrial pollutant emission levels [36]. Restricted by environmental constraints, paper-producing enterprises will seek to increase the efficiency of labor and capital factors, adopt more green production technologies, and reduce pollution emissions. Shi et al. (2021) found evidence that reorganizing capital elements is an important influence channel for pollution reduction by enterprises [37]. Stimulated by the EID policy, paper enterprises will reorganize the labor and capital input structure, increase high-end labor to improve production efficiency, save energy and reduce emissions, and increase capital investment in green production technology. Either through research and development or purchasing advanced production equipment, the pollution emissions of paper enterprises will be reduced. Technical effect is an important path for the EID policy to alleviate the pollution emissions of paper enterprises. Technology progress is the most effective way to solve the pollution emissions of enterprises. Technical effect has an indirect impact on the scale effect. The EID policy promotes the technological progress of green production of enterprises and alleviates the pressure of environmental regulation on the production scale of enterprises.

Exports positively impact the environmental effect of enterprises, consistent with the research conclusion of Su and Sheng (2021) [38]. This indicates that enterprises will be exposed to advanced production technology and stricter environmental protection standards when they export their products, thus reducing their pollution emissions. From the scale effect point of view, the export trade negatively impacts the scale of enterprises. The profitability of the international market is higher. Reducing domestic sales can reduce costs, which is a rational choice, leading to a decline in the overall GDP. In order to maintain more profit space, the enterprise will look for profit opportunities in the international market under the condition of limited production and make more profits. From the technical effect point of view, export trade can effectively improve the production technology of paper-producing enterprises. In the process of export trade, paper-producing enterprises can improve their technical level by introducing foreign advanced technology and equipment. This way can obtain higher output efficiency and a greener production process. Export trade has a positive effect on technical effect, consistent with the conclusions of Hettige et al. (1992) [39]. Export trade can not only obtain more market shares, but also use “learning by doing” to improve the technological level of enterprises. It not only alleviates the output problem caused by environmental regulations, but also accelerates the R&D and use of green technologies.

Import trade will increase the pollution of paper-producing enterprises, and the impact of China’s import trade on the environment is heterogeneous [40]. Although our study reached different conclusions from Su and Yang (2021) [41], it is in line with the idea of “hindering independent innovation” put forward by Ding and Zhang (2021) [42]. By importing raw materials or intermediate products with lower prices, paper-producing enterprises can increase their product profitability and have better market competitiveness. However, this will lead to the expansion of production scale and an increase in pollution emissions. From the scale effect perspective, import trade *lnim* can promote the enterprises’ gross production value. This shows that import trade is not conducive to enterprises reducing pollution through the scale effect. Import trade can increase their cost advantage and their profit margins; thus, it can promote the production scale of enterprises. From the technical effect point of view, import trade is not conducive to the technological progress of paper-producing enterprises. On the contrary, import trade will reduce enterprises’ production efficiency, mainly due to the “lock-in effect” of enterprises’ production and operation while using the international market [43]. The competitive advantage gained by enterprises operating in the international markets can also be very profitable, alleviating internal inefficiencies and reducing the motivation of enterprises to upgrade technology. This leads to insufficient investment in technology research and development, so the impact of import trade on technology effect is negative. Technical effect is an important way for enterprises to reduce pollution. However, import trade has not promoted the green production technology of enterprises as export trade, and has not realized the ideal situation of “market for technology”. The low price of raw materials in the international market will not only promote the progress of production technology, but also hinder the R&D of pollution reduction technology.

The EID policy has a positive moderating effect on the environmental effects of export trade. Enterprises will more actively use export trade under environmental regulation pressure to relieve their environmental resource pressure. Additionally environmental, regulation will make the environmental improvement effect of export trade more efficient. In terms of scale effect, the EID policy has a negative effect on export trade. This suggests that under the constraint of environmental regulation, enterprises will acquire foreign advanced technology and management through export trading. At the same time, the demand from the international market increases the production scale of enterprises. Although the production scale of the enterprise has increased, the increase in pollution emissions is not in linear proportion to the increase in scale. This is because the enterprise’s output increases with the improvement of the enterprise’s technical level, and the enterprise’s pollution emissions will not exceed the output growth. Regarding the technical effect, the EID policy has a positive moderating effect on export trade. This proves to some extent, the “trade innovation effect of environmental regulation” [21], consistent with the research findings and conclusions of Li and Chen (2013), and Shen and Zeng (2015) [44,45]. No obvious “trade diversion effect of environmental regulation” has been found in paper-producing enterprises [19]. Previous literature has found that the EID policy impacts the quality and markups of export products of enterprises [27,46]. The EID policy will speed up the pace of technology introduction in the process of export trade. In order to avoid environmental regulation and punishment, enterprises will look for advanced pollution reduction equipment in the international market and introduce management means that can improve production efficiency. The EID policy increases the environmental cost of enterprises, promotes product conversion, improves resource allocation efficiency, and improves the export products’ quality. To offset the additional costs brought by EID policy, enterprises will strive to improve the efficiency of resource allocation by improving product quality and reducing financing constraints [27]. This shows that the EID policy promotes production scaling and improves the technical level through export trade. EID policy also has a positive moderating effect on import trade. From the perspective of enterprise management, the pressure of the EID policy leads enterprises to reduce the production of intermediate products to alleviate environmental pollution and acquire them from the international markets. In terms of the scale effect, the EID policy has a negative regulating effect on import trade. Although increasing imports can result in a cost advantage, producing final products will still cause pollution. Under the constraint of environmental regulations, paper-producing enterprises will reduce their gross production value and production scale. No matter how much intermediate production can be saved by the import trade, the paper enterprises will have to process the final products, which will inevitably cause pollution emissions. The restriction of the final product scale by the EID policy leads to certain restrictions on the import scale of the intermediate products, and the EID policy will reduce the scale effect of the import trade. In terms of technical effect, the EID policy has a positive moderating effect on import trade. Stimulated by environmental regulation, enterprises gain a competitive advantage by increasing imports, which is not conducive to technological progress. This shows that implementing current environmental regulations is not conducive to enterprises’ upgrading and progress of paper-making enterprises through import trade. It is easier to choose import trade to avoid technological update. The enterprise saves intermediate production links by importing a lot of goods. The cost of avoiding the constraint of the EID policy by importing is far less than the cost of purchasing emission reduction technologies.

## 6. Conclusions and Policy Recommendations

### 6.1. Conclusions

Based on the Morgenstern theory and Porter hypothesis, this paper constructs the mechanism of EID policy and trade’s influence on enterprises’ environmental effects. Theoretically, EID policy and import and export trade can directly impact enterprises’ pollution emissions, and EID policy indirectly impacts enterprises’ pollution emissions through its regulating effect on trade. Then three hypotheses were put forward. Based on sample data collected from the China Industrial Enterprise Database, China Industrial Enterprise Pollution Database, and China Customs Enterprise Database, three hypotheses were validated by a PSM-DID regression approach. Based on the benchmark regression results, a parallel trend test and placebo test were conducted, respectively, and three robustness tests were adopted to ensure the robustness of the conclusions. The ACT theory influence mechanism was verified through scale, technical, and structural effects by replacing the explained variables. The specific conclusions are as follows:

(1) EID policy has a positive impact on enterprise pollution reduction. Enterprises subject to the EID policy will reduce pollution emissions to alleviate the cost increase.

(2) Export trade has a positive impact on enterprise pollution reduction. In the export trade process, by introducing and adopting advanced production technology and implementing more scientific management methods, the enterprise’s production efficiency can be greatly improved, the resource consumption can be reduced, resulting in pollution reduction.

(3) Import trade negatively impacts the pollution reduction of enterprises. In the process of import trade, enterprises gain a cost advantage through international markets. This cost advantage increases profit margins, and market competitiveness locks enterprises in a fixed productivity level. It increases the production scale, which leads enterprises to increase pollution emissions into the environment.

(4) EID policy has a positive moderating effect on both export trade and import trade. Export trade can reduce the pollution emissions of enterprises, but import trade can aggravate them. Environmental regulations have an impact on export trade. Under the constraints of environmental regulations, enterprises are more eager to find advanced production technology and management means through export trade. At the same time, enterprises may avoid the constraints of environmental regulations by purchasing more intermediary products from the international markets.

(5) EID policy mainly manifests its effect through the scale and technology effect, export trade through the scale, technology, and structure effect, and import trade through scale and technology effect. The moderating effect of environmental regulation on import and export trade mainly works through scale effect and technical effect. It is not easy for paper-making enterprises to change their product structure within a short timeframe.

### 6.2. Policy Implications

This study introduces environmental regulation into the discussion of the trade environment effect through the PSM-DID research method, and enriches the trade research theme of paper enterprises. The conclusion is of major significance to the formulation of China’s environmental and trade policies:

First, according to the different industry characteristics, different environmental regulation policies should be formulated. Some industries mainly cause air pollution, while others cause mainly water pollution. Targeted environmental regulation policies should be formulated instead of unified models, which should be evaluated as different categories.

Secondly, effective environmental regulation policies should be promoted in time, and regions should gain knowledge from each other. Environmental problems have strong negative externalities; thus, joint actions between different regions are needed for good results to be achieved. The central government should further strengthen the assessment weight of environmental problems when assessing the performance of local governments, promote “race to top competition,” and strictly guard against the emergence of “race to bottom competition.”

Thirdly, the government should focus more on the impact of trade on the environment, adjust the industrial and trade policies in time, and make use of the international market and advanced technologies of developed countries to improve the production efficiency of China’s paper-producing enterprises continuously. As paper-producing enterprises are capital-intensive, it can alleviate the financing pressure through green financial services and help enterprises to innovate in technology. At the same time, it attracts the capital and technology of developed countries using taxation and constantly eliminates the backward production capacity of China’s paper-producing enterprises.

Finally, formulating and implementing environmental regulation policies should consider the negative impact of trade. Although the existing environmental regulation can mediate import trade to a certain extent, leading to a positive impact on enterprise pollution reduction through scale effects, the intensity of this positive impact is very low. For example, although import trade alleviates the consumption of forest resources in China, it stimulates the increase of production scale. Moreover, its price advantage leads to enterprises’ lack of technological innovation motivation. Enterprises can completely transfer the cost to downstream consumers through pricing and don’t adopt clean technologies. The restriction objectives of environmental regulation should be not only at the scale of enterprise pollution emissions but also the direct promotion of environmentally friendly technologies. We need to consider bringing the production efficiency and green technology adoption of enterprises into the scope of environmental regulation assessment. This way, the enterprises will not hinder the technological progress of paper-producing enterprises whether they use the world market for exports or import cheap raw materials in the international market.

## Figures and Tables

**Table 1:** Statistical description of the variables.

Variable	Definition	Mean Value	Standard Deviation	Minimum Value	Maximal Value
Total industrial water consumption	W	3,467,342	2.40 × 10^7^	0	1.27 × 10^9^
Industrial wastewater discharge	W1	1,205,904	2,928,763	0	6.39 × 10^7^
PITI	Index	10.5062	20.97199	0	85.3
Scale	Size	338.9909	595.403	8	17,066
Asset specificity	Spe	0.42983	0.20905	0.0001976	0.9975
Gearing ratio	De	0.5623	0.2482	0	1
Capital intensity	KL	268.2867	6529.82	0.018059	923,903.9
Total factor productivity	opacf	4.54033	0.848889	−1.04636	10.59908
Market-oriented index	MI	7.8388	2.675432	1.400491	15.89166
Industrial structure	II	1.2589	0.59773	0.1983951	11.40506
Number of college students	St	106,390.3	161,837.7	0.02	966,438
per capita GDP	PGDP	48,345.04	40,170.7	1805	499,285
Export trade	ex	5434.647	105,107.5	0	6,043,336
Import trade	im	17,050.07	166,957.8	0	6,177,950

**Table 2:** Benchmark regression results.

	(1)	(2)	(3)	(4)	(5)	(6)	(7)	(8)	(9)
	PSM-DID	PSM-DID	PSM-DID	PSM-DID	PSM-DID	PSM-DID	PSM-DID	PSM-DID	PSM-DID
	lnW	lnW	lnW	lnW	lnW	lnW	lnW	lnW	lnW
DID	−0.442 ***	−0.384 ***	−0.387 ***	−0.346 ***	−0.275 ***	−0.273 ***	−0.247 ***	−0.261 ***	−0.197 **
	(0.089)	(0.088)	(0.088)	(0.087)	(0.087)	(0.087)	(0.087)	(0.088)	(0.090)
lnex	−0.078 ***	−0.081 ***	−0.079 ***	−0.079 ***	−0.077 ***	−0.077 ***	−0.080 ***	−0.080 ***	−0.078 ***
	(0.019)	(0.019)	(0.019)	(0.018)	(0.018)	(0.018)	(0.018)	(0.018)	(0.018)
DID_lnex	−0.127 ***	−0.116 ***	−0.114 ***	−0.108 ***	−0.110 ***	−0.110 ***	−0.106 ***	−0.106 ***	−0.108 ***
	(0.037)	(0.036)	(0.036)	(0.035)	(0.035)	(0.035)	(0.035)	(0.035)	(0.035)
lnim	0.094 ***	0.095 ***	0.094 ***	0.039 ***	0.031 **	0.030 **	0.032 **	0.031 **	0.033 **
	(0.014)	(0.014)	(0.014)	(0.014)	(0.014)	(0.014)	(0.014)	(0.014)	(0.014)
DID_lnim	0.119 ***	0.112 ***	0.112 ***	0.102 ***	0.105 ***	0.105 ***	0.107 ***	0.107 ***	0.108 ***
	(0.023)	(0.023)	(0.023)	(0.022)	(0.022)	(0.022)	(0.022)	(0.022)	(0.022)
lnSize	0.609 ***	0.588 ***	0.575 ***	0.625 ***	0.665 ***	0.666 ***	0.658 ***	0.659 ***	0.654 ***
	(0.038)	(0.037)	(0.037)	(0.035)	(0.035)	(0.035)	(0.035)	(0.035)	(0.035)
lnSpe		2.670 ***	2.887 ***	0.887 ***	1.167 ***	1.166 ***	1.138 ***	1.152 ***	1.054 ***
		(0.215)	(0.218)	(0.242)	(0.242)	(0.241)	(0.241)	(0.241)	(0.244)
lnDe			0.836 ***	0.482 ***	0.673 ***	0.672 ***	0.672 ***	0.672 ***	0.667 ***
			(0.180)	(0.178)	(0.178)	(0.178)	(0.178)	(0.178)	(0.178)
lnKL				0.443 ***	0.360 ***	0.360 ***	0.361 ***	0.359 ***	0.366 ***
				(0.030)	(0.031)	(0.031)	(0.031)	(0.031)	(0.031)
opacf					0.404 ***	0.404 ***	0.395 ***	0.397 ***	0.389 ***
					(0.044)	(0.044)	(0.044)	(0.044)	(0.044)
time					0.002	0.002	0.002	0.002	0.002
					(0.001)	(0.001)	(0.001)	(0.001)	(0.001)
MI						0.057	0.122	0.097	0.092
						(0.160)	(0.160)	(0.163)	(0.163)
II							0.649 ***	0.684 ***	0.746 ***
							(0.144)	(0.145)	(0.146)
St								0.017	0.036 **
								(0.015)	(0.015)
PGDP									−0.320 ***
									(0.093)
_cons	9.726 ***	8.912 ***	8.560 ***	7.185 ***	5.133 ***	5.001 ***	4.430 ***	4.293 ***	7.612 ***
	(0.205)	(0.207)	(0.221)	(0.230)	(0.324)	(0.478)	(0.499)	(0.512)	(1.105)
r2_a	0.144	0.161	0.163	0.187	0.195	0.195	0.197	0.197	0.198
year	Yes	Yes	Yes	Yes	Yes	Yes	Yes	Yes	Yes
spatial	Yes	Yes	Yes	Yes	Yes	Yes	Yes	Yes	Yes
cluster	Yes	Yes	Yes	Yes	Yes	Yes	Yes	Yes	Yes

Note: *** and ** represent statisticl significance at the 1% and 5% propability levels, respectively.

**Table 3:** Differing Measurements of lnW.

	(1)	(2)	(3)
	PSM-DID	PSM-DID	PSM-DID
	lnW	lnW1	L.lnW
Index	−0.008 ***		
	(0.002)		
DID		−0.298 ***	−0.181 *
		(0.103)	(0.094)
lnex	−0.079 ***	−0.058 ***	−0.075 ***
	(0.018)	(0.021)	(0.023)
DID_lnex	−0.103 ***	−0.112 ***	−0.109 ***
	(0.035)	(0.037)	(0.041)
lnim	0.030 **	0.042 ***	0.033 **
	(0.014)	(0.016)	(0.015)
DID_lnim	0.117 ***	0.088 ***	0.108 ***
	(0.023)	(0.024)	(0.024)
Control variables	Control	Control	Control
r2_a	0.200	0.179	0.223
year	Yes	Yes	Yes
spatial	Yes	Yes	Yes
cluster	Yes	Yes	Yes

Note: ***, ** and * represent statistical significance at the 1%, 5%, and 10% probability levels, respectively.

**Table 4:** Test of influence mechanism.

	(1)	(2)	(3)
	PSM-DID	PSM-DID	PSM-DID
	lnPd	lnPol	lnPer
DID	0.004 *	−0.119 ***	0.002
	(0.002)	(0.044)	(0.003)
lnex	−0.001 *	−0.041 ***	0.001 **
	(0.000)	(0.009)	(0.001)
DID_lnex	0.001 *	−0.026 *	0.000
	(0.001)	(0.014)	(0.001)
lnim	0.001 ***	0.024 ***	0.000
	(0.000)	(0.007)	(0.000)
DID_lnim	−0.001 **	0.045 ***	−0.001
	(0.000)	(0.011)	(0.001)
Control variables	Control	Control	Control
_cons	0.805 ***	5.606 ***	0.123 ***
	(0.028)	(0.549)	(0.037)
r2_a	0.997	0.240	0.100
year	Yes	Yes	Yes
spatial	Yes	Yes	Yes
cluster	Yes	Yes	Yes

Note: ***, ** and * represent statistical significance at the 1%, 5%, and 10% probability levels, respectively.

## Data Availability

Not applicable.
